# The critical intersection of environmental and social justice: a commentary

**DOI:** 10.1186/s12992-021-00686-4

**Published:** 2021-03-25

**Authors:** Leslie Solomonian, Erica Di Ruggiero

**Affiliations:** 1 Canadian College of Naturopathic Medicine, 1255 Sheppard Avenue East, Toronto, Ontario M4S 1R6 Canada; 2 Dalla Lana School of Public Health, University of Toronto, 155 College Street, Room 408, Toronto, ON M5T 3M7 Canada

**Keywords:** Ethics, Global justice, Ecological collapse, Social justice, Environmental health

## Abstract

The global crises of ecological degradation and social injustice are mutually reinforcing products of the same flawed systems. Dominant human culture is morally obliged to challenge and reconstruct these systems in order to mitigate future planetary harm. In this commentary, we argue that doing so requires a critical examination of the values and narratives which underlie systems of oppression and power. We argue for the moral necessity of a socially just approach to the ecological crisis.

## Background

The combined collective experience of COVID-19 and amplified conversation about white supremacy have thrust the complexity of the twin crises of global ecological decline and social injustice into vivid relief. The forces that have contributed to and continue to perpetuate the devastation of the biosphere are the very ones that have caused deep harm to and stark inequities among humans. These crises are mutually reinforcing consequences of the same flawed systems. As Rauf and Wainwright and others have compellingly laid out, in order to effectively mitigate future planetary harm, it is necessary to challenge systems of oppression and power; and justly distribute rights, duties, and responsibilities; drawing on diverse epistemologies to do so [1]. The purpose of this commentary is to argue for a socially just approach to the ecological crisis and suggest principles for future research and action.

### Creation of the crises

Dominant current culture is the result of a long history of the narrative that natural and human resources exist for exploitation, commodification and control, and to fuel economic growth [2]. This story is underpinned by values of competition, privatization, consumption, anthropocentrism, and dominance of Eurocentric technoscientific epistemology [3]. These values and narratives have been perpetuated and enacted by the global elite (economic, political, social) to concentrate power and wealth, which necessarily requires oppression of the masses and the marginalized [1, 4]. Entire groups of people are deliberately framed as having less worth by and to the benefit of those with power, embodied in a litany of genocides, enslavement, and systematic oppression. Imperial and colonial practices continue to exploit land and people for material gain. Theft and privatization of commonly-shared resources allows for exploitation and oppression of populations who can no longer afford to access that which has been commodified [2].

The tremendous technoscientific “progress” of the twentieth century has improved health, longevity, and quality of life for an estimated 20–30% of the global population at the systematic expense of the rest [2]. It also comes at a devastating cost of resource extraction, toxic waste generation, and uncompensated carbon emissions. The Great Acceleration and neoliberal capitalism have exponentially amplified harm to humans, other beings, and the biosphere itself [2]. This ecological catastrophe compounds social injustice [1, 5]. Those that are disproportionately affected by climate change (through consequences such as floods, droughts, fires, and conflict) are the same who have been exploited, displaced, marginalized, and murdered to concentrate wealth for the elite, whose actions have further driven the degradation of the environment [6, 7]. Privatization, commodification and destruction of natural and social resources denies access to resources required for basic subsistence [2]. Even proposed technoscientific “solutions” to the climate crisis are commodified and profitable, further marginalizing those without capital.

The values that underscore dominant social, political and economic systems have become so deeply ingrained into our collective subconscious that they can be nearly impossible to see, much less critique. While these systems were deliberately designed to concentrate wealth, many who benefit remain ignorant of the consequences of exponentially-increasing human activity in the name of improving longevity and quality of life [8]. For some time, the social and ecological consequences have been distant (geographically and chronologically), deliberately kept invisible from those who benefit from these systems [1]. Propaganda machines perpetuate myths of infinite growth, “sustainable” development, consumerism and technoscientific progress as an avenue to happiness and evidence of success. However, using gross domestic product and wealth accumulation to measure success will always come at the expense of the planet and other humans Transgenerational privilege and transnational exploitation will always result in inequitable capacity to “succeed.” And while the growing middle class holds an equal right to the standard of living and quality of life enjoyed by those situated in wealthier positions [7], this aspiration reinforces the cycle of extraction and waste generation [6]. Entitling all current and future inhabitants of Earth to the same standard of living as the currently most privileged is fundamentally unsustainable [2, 8].

### Perceiving possibilities

That powerful human groups have collectively allowed planetary conditions to deteriorate to this degree has been framed as an ethical failure: those groups carry a geographic and chronologic debt for which amends appropriately need to be made [2]. The language of “failure,” however, is fraught with shame, which tends to be totalizing, finalizing and deserving of retribution. This can induce paralysis, particularly for those who have been ignorantly complicit. In contrast, corrective and preventative justice frameworks speak more to the concept of guilt, which is often described as a feeling of responsibility which can motivate action [4, 7]. This perspective also speaks to the importance of moving toward what Albrecht coined as the Symbiocene [9]; to collectively cultivate systems that promote social and ecological homeostasis.

Utilitarianism theory would suggest that the desired goal is to promote the common good, or happiness [7, 8]. Given that it is unsustainable to aim to elevate the standard of living of the majority of the world to meet that which is enjoyed by a few, systems must be designed to ensure all have access to the necessities for at least a minimum standard of well-being and avoidance of suffering [5, 7]. There are many technoscientific strategies that are intended to do this, and are promoted from a utilitarian perspective. However, intentions are irrelevant if the consequences are harmful, which can be difficult to predict and measure, especially in a complex global system [2]. Herein lies a key limitation of utilitarian theory: multiple outcomes can result from a well-intended action which do not necessarily align with maximizing overall good. If the intent, for example, of genetically modifying foods is to increase productivity but the consequences are the loss of soil health and of food sovereignty by small, local farmers, it is problematic. If the intent is to preserve “wild” spaces, but the consequences are the removal of populations from their traditional lands and practices, it is culturally genocidal.

A utilitarian approach is also limited in that it would be difficult to convince those with privilege (at an individual or societal level) to willingly cede it for the betterment of the whole, particularly in a culture underpinned by values of individualism and meritocracy, and in which harmful consequences of individual actions are often indirect. This also raises the challenge of how much capacity an individual in a higher-income setting has to opt out of a system that encourages behaviours that may increase happiness for some at the expense of others. Behaving from a utilitarian framework requires adoption of a value system that considers the suffering of others, both proximal and distal to ourselves, and an honest reflection on what is truly necessary for our own happiness. Given that those with privilege have been indoctrinated with the idea that wealth concentration and consumption is the key to happiness, despite ample evidence to the contrary, this may be a hard concept to sell.

However, the planetary health crises will not be mitigated by the same faulty systems that created them. Young offers a model of responsibility that considers the role and responsibility of both individuals and systems; one that holds responsible both knowing perpetrators of ecocide *and* those naively complicit in the systems that cause ecocide [10]. This model can provide a scaffolding to theorize ways in which structures themselve can be critically held liable for global injustice. Ultimately, however, the process of creating more equitable and sustainable systems must begin with collectively and transparently redefining values and narratives in order to determine what the utilitarian baseline of “happiness” is [8, 11]. This requires deliberate integration of diverse epistemologies and priorities (in contrast to clinging to a rigid Eurocentric model of capitalistic and technoscientific solutions) [4, 7, 11]. Mechanisms must be in place to mitigate and dismantle global hegemonic structures in order to limit the influence of those with power seeking to preserve the status quo [5]. Procedural mechanisms should be designed in all sectors (scholarship, healthcare, commerce, policy, etc.) to ensure that those with power are critically interrogating whose interests and voices are represented or lacking. Implementation of models that equitably distribute decision-making power can center marginalized voices [12].

All stakeholders must be represented in this process, which also include other beings and the Earth herself [2, 7, 11]. While it may be impossible for humans to ever adequately represent others (human or otherwise), it is exciting to see progressive actions that advocate for universal rights of all species and ecosystems, such as the granting of legal rights to rivers and forests in New Zealand, and recent lawsuits by youth against countries for inadequate movement on the climate crisis. In their recent dissertation, Rodeiro makes an in-depth argument for environmental transformative justice; a lens that “offers an opening for (re)examining and (re) conceptualizing our practices, habits, values, norms, and priorities toward nature; in that reparative and reconciliatory activities represent an opportunity for progressively departing from current destructive and exploitative treatments of nature, thereby achieving and promoting sustainable stewardship.” [13]

Foster et al. offer a portfolio of principles to guide an interdisciplinary approach to planetary healing that is rooted in morality and equity as opposed to a flimsy foundation of economic/political/scientific theory [14]. Many of these principles reflect a key tenet of most Indigenous traditions: to honour all parts of the biosphere for their worth beyond the economic. Taking only what we need, leaving enough for others both now and in the future, and stewarding what remains are the essence of the Seventh Generation value of many Indigienous nations [15], and speak to the importance of intergenerational and environmental justice [7]. This also places humans within the complex interdependent web as opposed to superior to or separate from it. Any sustainable system of governance and decision-making must center these values to challenge anthropo- and contempocentrism [15].

## Conclusion

The climate crisis is a complex moral catastrophe, composed of multiple reinforcing ethical dilemmas. Although many who benefit from unjust global systems are unconsciously complicit in perpetuating them, it would be an ethical failure to not act once one becomes aware of the existence and causes of harm [4, 7]. The willingness to challenge existing systems and to center justice will require tremendous humility and spirit of collaboration [7]. Building on previous critical scholarship in planetary health, ecohealth and environmental health ethics, we recommend that research attend to best practices of elevating marginalized voices and representation in all sectors, and psychological and social strategies of shifting collective narratives and values. It is equally critical to continue to build and disseminate the evidence for the harmful impacts of inaction on these fronts. Recent global events such as the reverberations of COVID-19, and the surge of awareness of and resistance to white supremacy provide a window of opportunity to reimagine our future, as well as evidence that global, radical, rapid cooperation is possible. Narratives rooted in values of universalism and solidarity are being propagated in the form of Building Back Better and Just Recovery for All. These narratives, consistent with long-oppressed pre-colonial values [15], can form the foundation for collective action toward a more just and sustainable global community. As the late Maya Angelou gently reminded us, “when you know better, do better.”

## Data Availability

Not applicable.
